# Mobile clinics routing and scheduling in the Witzenberg region of South Africa

**DOI:** 10.1371/journal.pone.0310086

**Published:** 2025-01-07

**Authors:** Hannah J. Callaghan, Linke Potgieter, Nadia Le Roux

**Affiliations:** 1 Department of Logistics, Stellenbosch University, Stellenbosch, South Africa; 2 Department of Health and Wellness, Cape Winelands District, Ceres, South Africa; Cyprus International University Faculty of Engineering: Uluslararasi Kibris Universitesi Muhendislik Fakultesi, TÜRKIYE

## Abstract

Despite much literature on operations research applied to various healthcare problems, impactful implementation in public healthcare is limited, which often results in allocative inefficiency. This article uses a mobile clinic routing and scheduling problem in the Witzenberg region of South Africa as a case study to demonstrate the improvement of implementation success through cross-disciplinary collaboration, and also to propose a new three-stage approach for modelling a mobile clinic problem that incorporates continuity of care, fairness, and minimisation of distance travelled. Mobile clinics are used in many countries to improve access to healthcare for rural communities. Decision makers must assign farms or villages to mobile clinics, and determine their monthly visit schedules. To improve implementation success, we follow a collaborative three-phased mixed-methods approach with healthcare professionals to improve workload balance, fairness, and transportation cost. During phase 1, qualitative and quantitative data are gathered through qualitative research methods. In phase 2, fairly distributed optimal routes and schedules are designed using a three-stage model that incorporates a multi-vehicle routing problem to determine daily routes, a knapsack problem to establish a fair allocation of these daily routes between different clinics, and another variation on the vehicle routing problem to determine the monthly visit schedule that minimises the distance between the last farm visited on each consecutive day in the case of having to return to a farm the next day. Different input parameter estimations result in different routes and schedules. In phase 3, AHP is performed with main decision makers to determine their preferred solution. Final routes and schedules are designed based on model results, AHP results, and contextual input from decision makers. In our case study, an improved workload balance, a 23% reduction in total distance travelled, and buy-in to implement the changes, were obtained.

## 1 Introduction

Even with much literature on operations research (OR) applied to routing and scheduling problems, there seems to be limited practical implementation and impact on everyday decision-making in the public healthcare setting. This is not unique to routing and scheduling in healthcare, but is a general problem highlighted in literature about OR applied to healthcare [1, 2]. Common challenges for effective OR modelling in healthcare includes limited availability of quality data, distrust and a lack of collaboration between OR modellers and stakeholders in the public healthcare setting, and different priorities in deciding what the critical issues are. In this paper, our aim is to demonstrate, using a mobile clinic routing and scheduling problem as an example, how implementation challenges can be reduced by following a three-phased approach wherein healthcare professionals are actively involved in the first and third phases. Qualitative research methods and soft OR are used during the scientific process to improve collaboration and communication between OR modellers and healthcare professionals. In addition, a new mobile clinic problem is described along with a unique three-stage model, that is more similar to a home healthcare (HHC) problem, compared to mobile clinic problems described in literature. In our approach, continuity of care, fairness between mobile clinics, and minimisation of overall distance travelled are incorporated. Mobile clinics thus act as foundation in this study, on which the benefit of practical implementation can be demonstrated.

As a result of transportation barriers, many rural communities do not have easy access to primary healthcare facilities, even though it is considered a primary human right [3]. Due to this, many avoid seeking simple healthcare as to avoid the significant distances that would need to be travelled [4]. In South Africa, for example, only 84% of the population has access to healthcare services provided by the government, and most of this healthcare is centred in urban areas [5]. In addition to the scarcity of facilities, there are pressing health needs for a growing South African population, which lie in the areas of HIV/AIDS, TB and child health care. One way that the Department of Health, as well as several non-profit organisations are attempting to mitigate this for rural communities, is the introduction of mobile clinics that provide basic healthcare to the population in areas of most importance, such as emergency management of chronic diseases, general adult and paediatric care, HIV testing and counselling [6]. This is not unique to South Africa, but is becoming a global trend, especially in countries with challenges in accessibility to healthcare facilities [7].

In a typical mobile clinic deployment setting, the aim is to provide healthcare to a general population. The mobiles are based at a local clinic (depot) and visit their respective assigned locations for the day, after which they either return to the local clinic, or travel to the next location. The mobile clinics do not go to specific farms, but rather get set up at a specific location and the population must travel a distance to reach the mobile clinic. The goal is to maximise the coverage and provide easier access to healthcare [8, 9]. Mobile clinics can provide routine general care or they can aid in critical situations such as natural disasters when the need for care is vitally important [10]. Some challenges in mobile clinic deployment include lack of funding, resources, infrastructure, permanent staff, as well as the lack of followed through planning [10]. In addition, the routing and scheduling of the mobile clinics are typically completed by healthcare professionals and often done manually, which often results in allocative inefficiency, despite spending a lot of time on the exercise. Given the limited public healthcare resources, this time could be spent more usefully on other activities [11].

Mobile clinic problems can be described as a vehicle routing problem (VRP), and depending on the specific problem, may include multiple depots, time-windows, or in the case of mobile clinics repeatedly assigned to the same location, using a periodic VRP (PVRP). A detailed review of PVRP literature is given by Campbell and Wilson [12] and Wang et al. [13]. A characteristic of a typical mobile clinic problem in literature, is that mobile clinics are generally assigned to location points randomly, so that continuity of care is neglected. In contrast to routing problems in general, continuity of care is an important and unique factor to consider in healthcare routing problems, since it improves the quality of care [4]. Rodriquez-Martin et al. [14] and Savaser and Kara [4] are of the few studies that incorporate continuity of care in the model so that the customers (or patients) are served by the same healthcare staff at all visits.

Along the same vein, HHC is a well studied topic in the OR literature. Fikar and Hirsch [11] and Cissé et al. [15] provide detailed discussions on HHC routing and scheduling problems. In a typical HHC setting, nurses are assigned to specific patients, and plan their daily work schedule accordingly. The nurses are based at a hospital and visit their assigned patients throughout their working day, after which they return to the hospital to prepare for the next day or perform any additional services at the hospital [16]. Their travel routes and the times of their arrivals each day have to be planned [11]. The nurse can either begin their route from their home or from one of many hospitals, resulting in the system having multiple depots. Multiple depots are often introduced when the service areas are large or not easily accessible [15]. There are various considerations that are modelled in a HHC problem. Most research focus on minimising the travel time or travel cost of the route [11, 15, 16]. Most commonly, the goal is to maximise the quality of care received by the patients while minimising extra criteria such as travel time or travel cost [15]. Additionally, there is an emphasis on the satisfaction of patients and staff. Satisfaction of patients means that the quality of care provided is at a high enough standard to meet the patients’ healthcare needs. This implies that the nurses should spend an adequate amount of time in providing healthcare to patients and are not rushed or limited in their resources. Satisfaction of nurses means that the workload is balanced [17], this satisfaction has a direct impact on the nurses’ level of service provided and consequently effects the satisfaction of patients [17]. Workload balancing ensures that there is an even spread of the work between the nurses [18]. This results in the nurses maintaining their motivation, as they feel that the work is equally spread and they are not having to carry a heavier burden than the other nurses. The HHC problem (and by extension the mobile clinic problem) can also be described as an extension of a VRP [19, 20], wherein a fleet of vehicles must deliver products to a set of customers, after which the vehicles must return to the depot [21]. The VRP is extended by adding constraints that are specific to the context of the HHC problem [15]. Here the nurses (mobile clinics) are seen as the vehicles and the patients (farms) as the customers. By using a VRP formulation, routes and schedules can be optimised in terms of both cost and workload balance.

The mobile clinic setting addressed in our study is in the Witzenberg region, located in the Western Cape in South Africa. It is a mountainous area that is mostly comprised of farm land, and contains only eight healthcare facilities and one district hospital. These factors make it difficult for the population to access care. Three mobile clinics have been deployed into the case study region, to provide healthcare to the farming communities. There are 148 farms, with population numbers ranging from 10 to over 1000. Each mobile clinic has an assigned route which services specific farms. There are two main requirements that should be adhered to to reduce communication barriers and provide continuity of care on the farms. First, the time between two consecutive visits to a specific farm should always be the same, so as to create a predictable schedule that is easy to remember for the farm workers. Second, the farms should be visited by the same mobile clinic. The mobile clinics are based at two clinics in the region and are serviced by staff from these clinics. Once they have completed visiting the assigned farms for the day, they are expected to provide their services in the clinic. In cases where they can’t complete the work on the assigned farm on the day, they return the next day. The routes and schedules for the mobile clinics in the Witzenberg region were created years ago and the efficiency and fairness of these routes between the mobile clinics are under scrutiny. Routes are overlapping between mobile clinics, and the workload is not evenly spread between the mobile clinics, where some mobile clinics are serving larger farming communities than others (and hence more patients), and some mobile clinics are covering longer distances but serving smaller communities.

The HHC setting lines up with the description of the mobile clinics in the Witzenberg region where mobile clinics visit specific patients (in our case, farms) on a determined route each day. Since the Witzenberg mobile clinics can be seen as a HHC setting, much of the factors considered in HHC models are informative to our study, although there are some differences. Continuity of care should be adhered to, similar to the study by Savaser and Kara [4]. Mobile clinics in Witzenberg visit a farming community at the same time slot at each visit, hence this situation differs from the study by Savaser and Kara [4] in its periodicity of the visits (visit frequencies and rules), and thus patient time windows are not applicable. However, they have to start and return to the same location every day, which is similar to the HHC setting but differs from the study by Savaser and Kara [4]. Finally, in the HHC problem, there is no need for nurses to return to previous day patients as in our case study.

The aim of this paper is two-fold: (1) to demonstrate how mixed-methods research can facilitate collaborative work and communication between OR academics and healthcare professionals to impact decision-making in healthcare, and (2) to present a unique three-stage model that combines two variations of the VRP and a knapsack problem to minimise overall travel cost and workload imbalances. This collaborative approach results in a solution that is more considerate of the practical healthcare context (and associated constraints), the satisfaction of healthcare staff and the quality of care of the patients, and takes into account the possibility of having to return to farms already visited, which is not something previously considered on literature. In this study, the aim was to feasibly improve the workload balance, fairness, and transportation cost of the mobile clinics in the specified region.

The paper is organised as follows. Section 2 provides an overview of the qualitative research phase used in this study referred to as phase 1. Section 3 presents phase 2, in which the model is defined, and results from this phase are given. Section 4 presents the results of the soft OR phase (phase 3) during which model results were analysed and adjusted. This is then followed by a discussion in Section 5. Finally, the conclusions as well as possible future research avenues are provided in Section 6.

## 2 Phase 1

During phase 1, qualitative research techniques were used to do background research, identify the problems and gather a wide range of qualitative and quantitative data. Primary data included contextual information, ideas and opinions expressed by staff during frequent conversations and unstructured interviews, a focus group interview, a questionnaire, and personal observations by the author during a day travelling with the mobile clinic. Secondary data included the current route maps, schedules, vehicle travel logs for the period January 2023 to April 2023 and daily patients statistics received from the clinic for the period May 2022 to April 2023. These raw data had to be converted into a format that is easily useable for modelling. Ethics was approved on 3 April 2023 by the REC:Social, Behavioural and Educational Research Ethics Committee at Stellenbosch University under project ID number 27301.

### 2.1 Primary data collection process

A number of in-person and online meetings were scheduled between the three authors, two of which are operations researchers, and the third a doctor formally appointed to work with the Witzenberg mobile clinics, as a healthcare partner. Initially, the mobile clinic problem was presented to us by the doctor in an attempt to find a better long-term solution. We therefore had both a strong champion, and a critical issue, which are two of the main elements identified by Carter and Busby [2] to increase implementation success. These meetings aided as a platform to discuss context, viewpoints and progress. Information was extracted from these meetings to identify important focus areas. These meetings were important to remain on the same page in terms of priorities, feasibility, and grow in mutual understanding and trust.

Permission was obtained from the Department of Health in the Western Cape, Witzenberg region, to interview healthcare staff associated with the mobile clinics. Healthcare staff were identified and recruited during the period 26 April to 16 May 2023 for a 2-hour focus group interview that was conducted on 17 May 2023 to improve our understanding of the problem from their perspective. Interviewees were invited per email as well as in-person, and were aware of the date and purpose of the interview well in advance. Six healthcare staff members assigned to the mobile clinics, either in a managerial role or actively working on the clinics, attended the interview. In addition, they completed a questionnaire after the interview, from which we obtained insight into personal preferences by thematically analysing both the focus group interview and questionnaire. Written consent was obtained from participants to use anonymised data from the interview and questionnaire. A few days after the interview, a day was spent on one of the mobile clinics to get more contextual information and practical exposure to how the mobile clinics function on their day-to-day operations.

### 2.2 Primary data results

From the information gathered during the interview, insights and preferences from staff could be understood, informing the model development in phase 2. It gave the opportunity for staff to voice their opinions and feelings about their work, building trust in the process. The need to rearrange the schedule to allow for less wasted time travelling and more time to see patients was emphasised. Some of the important observations identified through analysis include: (1) a general feeling that some mobile clinics work longer hours due to more patients needing to be seen, or they were required to travel further, compared to other mobile clinics; (2) a need for schedules to not be based on staff performance, but to focus on fairness in workload allocation, for a longer term solution (i.e. in the case of changing staff, there should not be the need to rearrange routes and schedules); (3) the importance of continuity of care (the same mobile clinic visiting the same farms every month), as the general consensus was that it takes two years to build trust with farm managers and patients; (4) the challenges in workday planning due to seasonal farm workers and the stochastic nature of the healthcare services and number of patients seen per farm; (5) different viewpoints about the feasibility of an appointment system for the mobile clinics to improve planning; (6) a need for extra staff in the local clinic (instead of the mobile clinics) as they still service the majority of the population, however, the feeling was that staff struggle to work in the local clinic after a day on the mobile clinics, as the travelling from farm to farm is enervating; (7) preference in the order of farms visited, for example, completing smaller areas within the region during consecutive days, and two long days (large farms) being spaced apart with less busy days in between.

**Fig 1:**
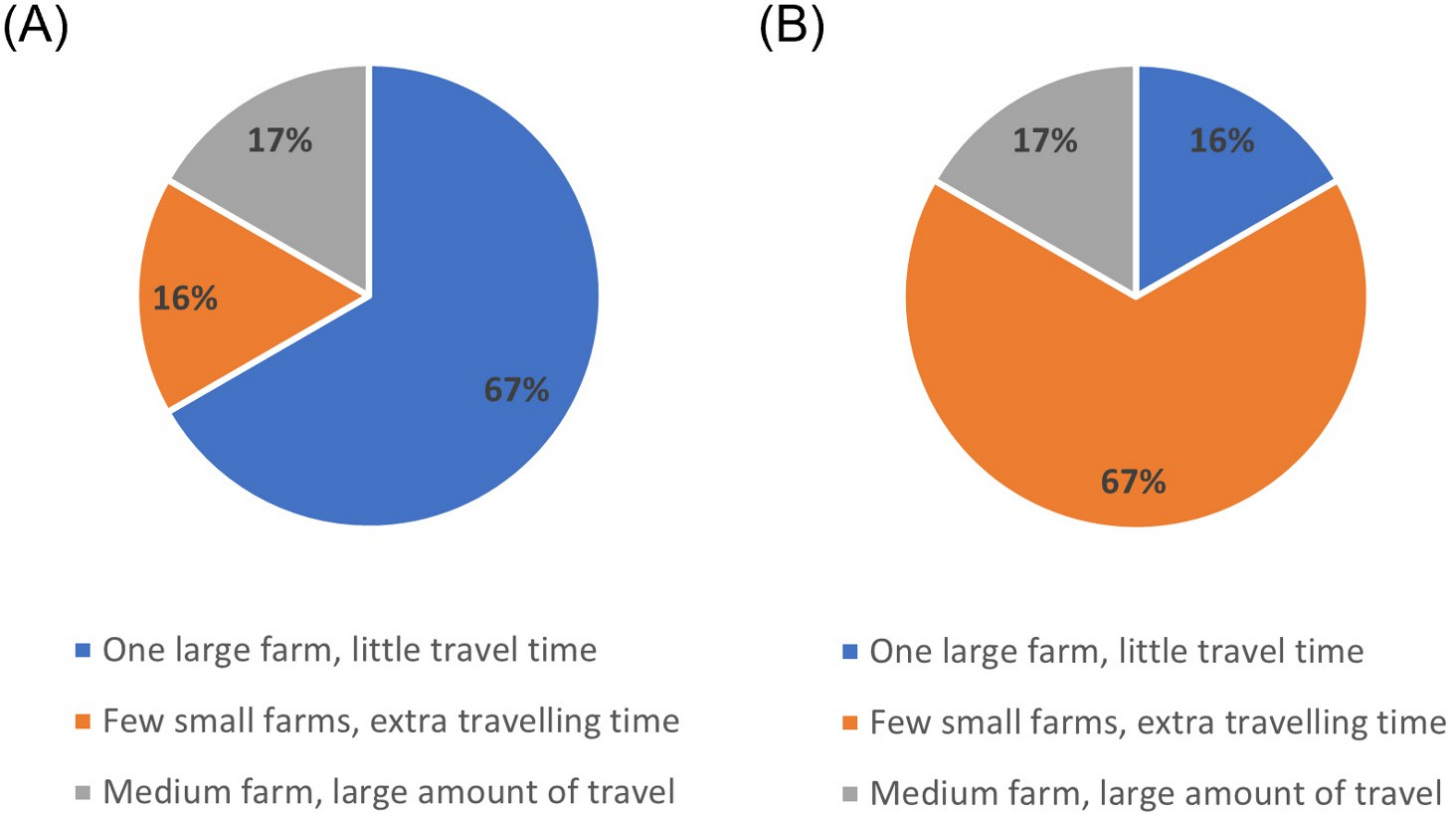
Questionnaire results about work demands. Results from the questionnaire about (A) most demanding work on the mobile clinic and (B) least demanding work on the mobile clinic.

An important observation from the questionnaire is that 67% of participants find visiting large farms to be the most demanding work, seen in Fig 1, however it is also seen by the participants as the most preferred work, as the majority of time would be spent on providing healthcare services, instead of travelling and packing up between farms. Equal distribution of large farms is therefore important to increase overall job satisfaction amongst healthcare staff.

### 2.3 Secondary data

Data was obtained from the Western Cape Department of Health, some in electronic format and some as hard copies, during the period Feb 2023 to Jul 2023. None of the data received include sensitive or personal information of staff or patients. In this process, the support of a strong champion cannot be overemphasized, as data was not always easy to obtain. Systems are not integrated, hence data was obtained from different administrative staff. Even identifying from whom the data could be requested, would not have been possible without a ‘champion’.

Information about the current routes and monthly schedules were provided, including the farms that were visited by each of the mobiles. Using this information, a complete distance and travelling time matrix were constructed, containing distances between each of the farms and depots. Schedules indicated that four days per week are used for visiting the farms, making sure that each farm is visited at least once a month, with the fifth day of the week reserved to complete admin and work in the local clinics, or alternatively return to a farm if there were too many patients on a particular day.

The mobile staff provided the total number of patients seen each day by the mobiles for the period May 2022 to April 2023. This data was used to determine the average number of patients seen on each day of the schedule, and this was then linked to the list of farms seen on that specific day in the schedule. Farm specific data was not available, and the average number of patients per farm on a specific day was used as an estimate instead. The average standard deviation of the service times for the routes was 40.72. Some routes had a standard deviation of over 80, however this large standard deviation can be explained due to these routes having large numbers of seasonal workers only present during some months. Where there appeared to be abnormalities in the data, such as missing values or outliers, they were dealt with individually by asking the mobile staff to account for these values, if no answer could be given then these values would be excluded from the averages.

**Fig 2:**
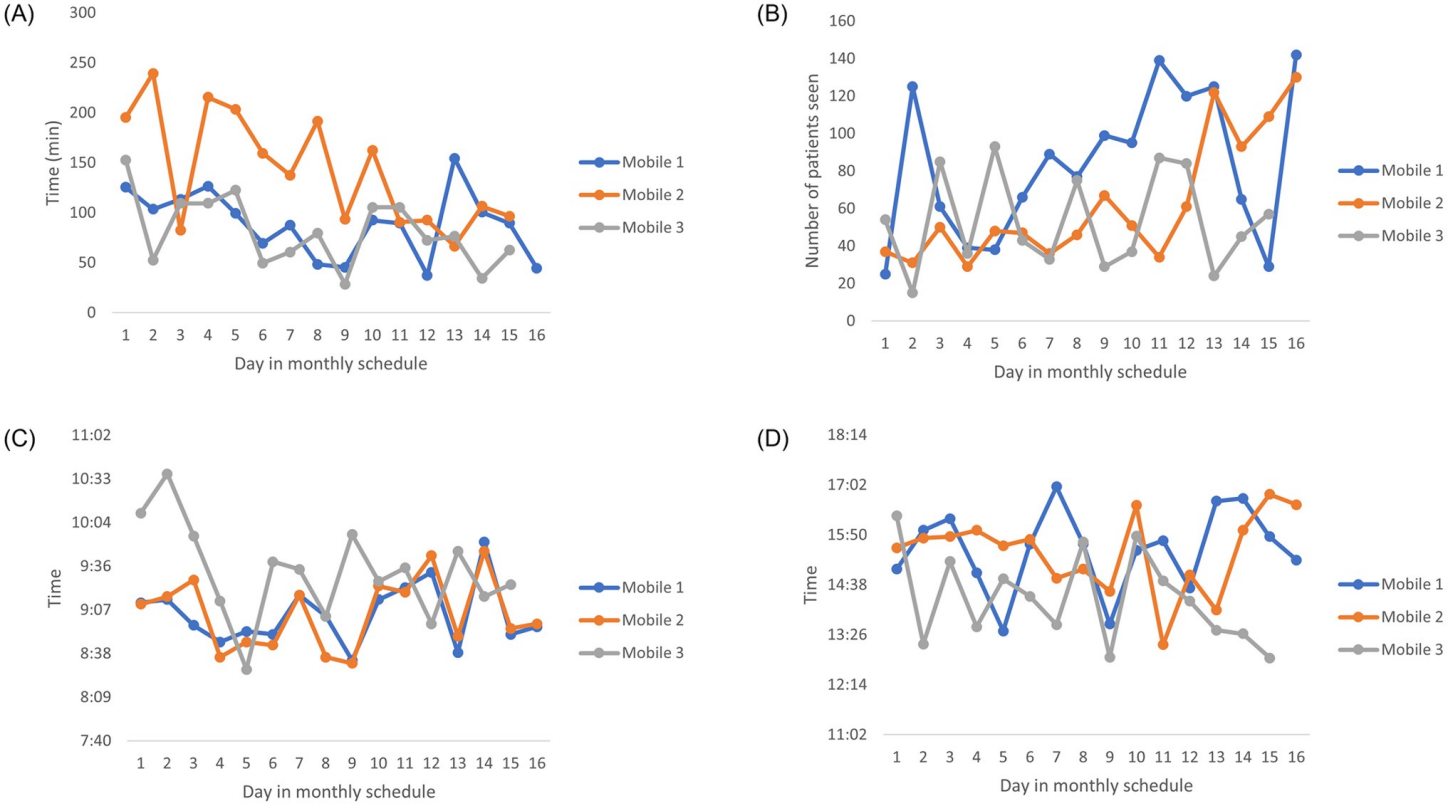
Patient and travel data for the month of February 2023. Data for the month of February 2023 depicting (A) time spent travelling for the three mobiles, (B) the number of patients seen by each mobile, (C) the times the three mobiles left the depot and (D) the times the three mobiles returned to the depot for each day of their schedule.

Additionally, the mobile clinics travel log was provided. This contained information such as how far they travelled between each stop, the length of each stop as well as the time the mobile left and returned to the depot. The travel logs indicate a clear workload imbalance between the mobile clinics (see Fig 2 illustrating travel log data for February 2023). Fig 2(A) shows the travel time of each of the mobiles. Mobile 2 spends a lot longer travelling compared to the other mobiles. Fig 2(B) shows the number of patients seen by each of the mobiles. Again there are major differences between the mobiles, with some mobiles providing care to a large majority of patients. Fig 2(C) and 2(D) shows the start time and end time of the mobiles for each day of their schedule in February. Mobile 3 starts later and ends earlier on most days which results in a much shorter day of work. The travel log combined with the monthly patient data was used to determine the average length of time that a staff member would spend with a patient.

## 3 Phase 2

During phase 2 of the research, OR techniques were used to develop a mathematical model, run different experiments and find corresponding optimised routes and schedules for the different mobile clinics.

A three-stage model is presented in this section to solve the Witzenberg mobile routing and scheduling problem, wherein each stage prioritises a different goal. Data was extracted from phase 1 and used in the development and execution of phase 2. There are 148 farms that require visits once a month, by one of the three mobile clinics. Constraints are introduced into stage 1 of the model to ensure that all the farms are visited by only one of the mobile clinics. As established from the focus group interview, these farms must be seen by the same mobile clinic each month to ensure continuity of care. By creating a schedule for each of the mobile clinics that repeats itself monthly, results in each farm being seen by the same mobile each visit.

### 3.1 Modelling considerations and input parameters

#### 3.1.1 Service times

Each farm requires a certain length of time from the mobile clinic, based on the number of people residing on the farm, and the size of the farm itself, as multiple stops are in some cases required to ensure easy access to the entire population on the farm. In the case of a deterministic model, service times used per farm should implicitly incorporate the inherent stochasticity in the number of patients seen each month per farm. With the data provided, the service time per farm is determined using multiple methods. For each method, alternative schedules are determined.

The first method for determining the service times, is to take the average number of patients seen every month on a farm and multiply this with the average service time per patient across all the farms, with an additional buffer time of an extra 10% of the calculated service times. This method is referred to as E1. Additional methods to determine the service times, are to take the average length of time spent on a farm every month and add extra buffer time. The extra buffer time is calculated either to be an additional 10% of the calculated service time or the standard deviation of the average length of time spent on a farm. These methods are referred to as E2 and E3, respectively. Finally, the service times can be taken as the upper bound on time spent per farm from historical data, calculated using the maximum number of patients seen by the mobile clinic on a farm and multiplying this value by the average time taken to service each patient, as calculated in the first method. This method is referred to as E4.

#### 3.1.2 Travel time

The travelling distance and travelling times between each farm and/or depot were determined previously. For stage 1 a central depot is used. This is due to it being the depot of two of the three mobiles, and the third mobile is stored not too far away at another depot. In stage 3, the distances that are used are the distances from the last farm visited on a day to the last farm visited on the next day. This is to consider minimum travel time between routes on consecutive days in the case of next day follow up visits when all patients could not be seen on the scheduled day. Typically, the mobile staff would first finish their daily route, and then revisit a previous day’s unfinished farms.

The travel times that are used are based off the values obtained from Google Maps. These travel times do not include traffic or slower travel due to bad weather conditions. An accurate long-term prediction of these factors is not feasible, thus they cannot be incorporated into the model. Instead, a buffer time of 30 minutes is allowed so that any additional time spent will not cause for the staff members to return to the clinic past the end of their workday.

#### 3.1.3 Length of routes and schedule

The total service time and travel time combined for each daily route should not exceed an 8-hour work day. For each of the three mobiles, it would be ideal to schedule daily routes over a 16-day time horizon, as this allows for 4 workdays of admin per 4-week cycle. Thus, for the three mobiles, 48 daily routes are to be determined. If the number of daily routes obtained by the model is less than 48 days, this would allow for extra time for admin during busy months as well as allowing time for the staff to work in the clinics.

#### 3.1.4 Fairness

From the focus group interview, fairness is viewed as having an even distribution of similar tasks or activities between each of the mobile clinics. Thus, each mobile clinic should have a similar total time spent with patients and spent travelling each month. This has been incorporated into stage 2’s constraints. The absolute value difference in the total service times allocated to each mobile clinic is set to be within a small difference. The same is incorporated for the time spent travelling. An additional method to ensure fairness between the mobile clinics is to evenly distribute the routes containing large farms and small farms. Routes with large farms are very tiring on an emotional level, due to the large number of patients seen. Routes with several small farms on the same day are seen as less demanding. Routes with large farms are defined to be routes that contain only 1 or 2 farms. The distribution of large and small farm routes is incorporated into a constraint in stage 2 to ensure that the difference in the number of large and small farm routes assigned to each mobile does not exceed one.

#### 3.1.5 Workload balance

Within each mobile there needs to be balance in the daily schedule, as well as predictability for their routes. This was incorporated in stage 3. Once the routes have been fairly assigned to each mobile, a balanced schedule should be created. Balance is seen as a schedule that does not have large farms on consecutive days and ensures that consecutive routes are in a similar area. This is introduced by assigning penalties to consecutive large farms, resulting in this connection to not be included. Since we are dealing with distances and constructing an order for the routes to be visited, a VRP can be used, with each route being seen as a node.

### 3.2 Stage 1

During stage 1, multiple daily routes are created, with all farms included in one of the daily routes to be travelled by one of the three mobile clinics. This is modelled as a multi-vehicle routing problem with a central depot and time windows. Each of the farms have a service time that must be adhered to, ensuring that all patients are seen within the mobile clinic’s visit.

#### 3.2.1 Stage 1 sets, parameters, variables and objective function

The model uses sets to define the structure of the problem, where

$$\begin{array}{ccc} F & \;\;\text{is}\;\text{the}\;\text{set}\;\text{of}\;\text{all}\;\text{farms,}\;\text{with}\;\text{a}\;\text{cardinality}\;\text{of}\;f, & \\ D & \;\;\text{is}\;\text{the}\;\text{set}\;\text{of}\;\text{depots,}\;\text{with}\;\text{a}\;\text{cardinality}\;\text{of}\;e, & \\ N & \;\;\text{is}\;\text{the}\;\text{set}\;\text{of}\;\text{all}\;\text{nodes,}\;\text{which}\;\text{includes}\;\text{all}\;\text{the}\;\text{farms}\;\text{as}\;\text{well}\;\text{as}\;\text{the}\;\text{depot,}\;\text{thus}\; & \\ & \;N=F\cup D\text{,}\;\text{which}\;\text{has}\;\text{a}\;\text{cardinality}\;\text{of}\;n=f+e,\;\text{and} & \\ V & \;\;\text{is}\;\text{the}\;\text{set}\;\text{of}\;\text{all}\;\text{vehicles,}\;\text{with}\;\text{a}\;\text{cardinality}\;\text{of}\;v. \end{array}$$

Here *f* is 148, *e* is 1, *n* is 149 and *v* is 48.

The model uses several constant parameters for which the values are obtained from the data provided in phase 1, where

$$\begin{array}{ccc} s_{i} & \;\;\text{is}\;\text{the}\;\text{service}\;\text{time}\;\text{of}\;\text{farm}\;i, & \\ {}[a_{i},b_{i}] & \;\;\text{is}\;\text{the}\;\text{time}\;\text{window}\;\text{of}\;\text{farm}\;i, & \\ d_{ij} & \;\;\text{is}\;\text{the}\;\text{travelling}\;\text{distance}\;\text{between}\;\text{farm}\;\text{or}\;\text{depot}\;i\;\text{and}\;j, & \\ v & \;\;\text{is}\;\text{the}\;\text{number}\;\text{of}\;\text{vehicles,}\;\text{and} & \\ t_{ij} & \;\;\text{is}\;\text{the}\;\text{travelling}\;\text{time}\;\text{between}\;\text{farm}\;\text{or}\;\text{depot}\;i\;\text{and}\;j. \end{array}$$

There are two decision variables defined for stage 1. The first is *x*_*ijk*_, which is used to indicate the path between farm or depot *i* and *j* on route *k*, hence

$$\begin{array}{c} x_{ijk}=\{\begin{array}{cc} 1; & \text{if}\;\text{mobile}\;k\;\text{travels}\;\text{between}\;\text{farm}\;\text{or}\;\text{depot}\;i\;\text{and}\;j\\ 0; & \text{otherwise}. \end{array} \end{array}$$

The second variable is *y*_*ik*_ which is used in indicating the time at which mobile *k* arrives at farm *i* to begin its service. This value of *y*_*ik*_ must be within the time window of service for the farm, then

$$\begin{array}{c} y_{ik}\;\text{is}\;\text{the}\;\text{starting}\;\text{time}\;\text{of}\;\text{serving}\;\text{farm}\;i\;\text{with}\;\text{vehicle}\;k,\;\text{and}\;y_{ik}\in \left[a_{i},b_{i}\right]. \end{array}$$

The objective function of stage 1 is to minimise the total distance travelled across all daily routes.

#### 3.2.2 Stage 1 formulation

By using all the defined sets, variables, parameters and the objective function, the multi-vehicle routing problem with time windows, is given by

$$\begin{array}{cc} \text{Min}\;\sum_{k=1}^{v}\sum_{j=1}^{n}\sum_{i=1}^{f}d_{ij}x_{ijk}, & \end{array}$$ (1)

$$\begin{array}{cc} \text{subject}\;\text{to:}\;\sum_{i=1}^{n}\sum_{k=1}^{v}x_{ijk}=1, & j\in N,j\neq 1 \end{array}$$ (2)

$$\begin{array}{cc} \sum_{j=1}^{n}\sum_{k=1}^{v}x_{ijk}=1, & i\in N,i\neq 1 \end{array}$$ (3)

$$\begin{array}{cc} \sum_{j=1}^{f}x_{ijk}\leq 1, & k\in V,i\in D \end{array}$$ (4)

$$\begin{array}{cc} \sum_{i=1}^{f}x_{ijk}\leq 1, & k\in V,j\in D \end{array}$$ (5)

$$\begin{array}{cc} \sum_{i=1}^{n}x_{ijk}=\sum_{i=1}^{n}x_{jik}, & k\in V,j\in F \end{array}$$ (6)

$$\begin{array}{cc} a_{i}\leq y_{ik}, & k\in V,i\in F \end{array}$$ (7)

$$\begin{array}{cc} y_{ik}+s_{i}\leq b_{i}, & k\in V,i\in F \end{array}$$ (8)

$$\begin{array}{cc} x_{ijk}\left(y_{ik}+s_{i}+t_{ij}-y_{jk}\right)\leq 0 & k\in V,i,j\in F \end{array}$$ (9)

$$\begin{array}{cc} x_{ijk}=0, & k\in V,i,j\in D \end{array}$$ (10)

$$\begin{array}{cc} x_{ijk}\in \{0,1\} & k\in V,i,j\in N \end{array}$$ (11)

Objective function (1) minimises the total routing cost, which is equivalent to minimising the travelling time. Eqs (2) and (3) ensure that each farm is visited only once and each mobile leaves the farm only once. Constraints (4) and (5) result in there being no route that does not originate from a depot. Constraints (6) ensures that there is route continuity, if a vehicle arrives at a farm, it must leave the farm before continuing with the route. Constraints (7) and (8) ensure that the visits to the farm are within the time window. Constraint (9) establishes the relationship between the vehicle departure time from a farm and its immediate successor, and constraint (10) results in there being no routes between the depots. Constraint (11) ensures that *x*_*ijk*_ is only ever either a 0 or 1.

### 3.3 Stage 2

Stage 2 uses the routes determined in stage 1 and allocates them to a specific mobile clinic, with the aim of even distribution of the workload between the mobile clinics. The routes have the following attributes: the number of farms visited, the total distance travelled from the central depot, the total service time to all the patients seen on the route, as well as if it is a route in which large farms or small farms are seen. Since in stage 1, the distances are determined to the central depot, for stage 2 new distances must be determined for each of the routes to each of the depots. The set of the routes used in stage 2 corresponds to the routes that were created by each of the vehicles in stage 1. Using this information, we can produce a formulation to assign the routes to the mobile clinics.

#### 3.3.1 Stage 2 sets, parameters, variables and objective function

The model uses sets to define the structure of the problem, where

$$\begin{array}{ccc} R & \;\;\text{is}\;\text{the}\;\text{set}\;\text{of}\;\text{all}\;\text{routes,}\;\text{with}\;\text{a}\;\text{cardinality}\;\text{of}\;r\text{,}\;\text{and} & \\ W & \;\;\text{is}\;\text{the}\;\text{set}\;\text{of}\;\text{the}\;\text{mobiles}\;\text{clinics,}\;\text{with}\;\text{a}\;\text{cardinality}\;\text{of}\;w\text{,}\;\text{and} & \\ E & \;\;\text{is}\;\text{the}\;\text{set}\;\text{of}\;\text{all}\;\text{depots}. \end{array}$$

Stage 2 takes in several constant parameters which are defined after stage 1 was run, where

$$\begin{array}{ccc} p_{g} & \;\;\text{is}\;\text{the}\;\text{total}\;\text{service}\;\text{time}\;\text{of}\;\text{all}\;\text{the}\;\text{farms}\;\text{on}\;\text{route}\;g, & \\ m_{gh} & \;\;\text{is}\;\text{the}\;\text{total}\;\text{distance}\;\text{travelled}\;\text{on}\;\text{route}\;g\;\text{which}\;\text{is}\;\text{serviced}\;\text{by}\;\text{mobile}\;h, & \\ L_{g} & \;\;\text{equals}\;\text{1}\;\text{if}\;\text{a}\;\text{large}\;\text{farm}\;\text{is}\;\text{seen}\;\text{on}\;\text{route}\;g\text{,}\;\text{else}\;\text{equals}\;\text{0}, & \\ S_{g} & \;\;\text{equals}\;\text{1}\;\text{if}\;\text{more}\;\text{than}\;\text{6}\;\text{small}\;\text{farms}\;\text{are}\;\text{seen}\;\text{on}\;\text{route}\;g\text{,}\;\text{else}\;\text{equals}\;\text{0} & \\ \lambda & \;\;\text{is}\;\text{a}\;\text{tolerance}\;\text{variable}\;\text{for}\;\text{the}\;\text{total}\;\text{service}\;\text{time,}\;\text{and} & \\ \tau & \;\;\text{is}\;\text{a}\;\text{tolerance}\;\text{variable}\;\text{for}\;\text{the}\;\text{total}\;\text{travel}\;\text{distance}. \end{array}$$

The values for *r* is equal to *v*, *w* is 3, λ and *τ* were determined through sensitivity analysis and were both determined to be 25. For stage 2, *z*_*gh*_ is used to indicate whether a route *g* is travelled by mobile *h*, by defining

$$\begin{array}{c} z_{gh}=\{\begin{array}{cc} 1; & \text{if}\;\text{route}\;g\;\text{is}\;\text{travelled}\;\text{by}\;\text{mobile}\;h\\ 0; & \text{otherwise}. \end{array} \end{array}$$

The objective function for stage 2 is to evenly distribute the workload between the mobile clinics while ensuring that the service time to travel time ratio is maximised so that the staff spend most of their time providing healthcare to patients rather than travelling between the farms. This can be achieved by maximising the sum of the ratio of service time to travel time for each of the mobile clinics [9].

#### 3.3.2 Stage 2 formulation

With the defined objective function, sets, parameters and variables, the formulation is then

$$\begin{array}{cc} \text{Max}\;\sum_{h=1}^{w}\sum_{g=1}^{r}\frac{p_{g}z_{gh}}{m_{gh}} & \end{array}$$ (12)

$$\begin{array}{cc} \text{subject}\;\text{to:}\;\sum_{h=1}^{w}\sum_{g=1}^{r}z_{gh}\leq \lceil \frac{r}{w}\rceil & \end{array}$$ (13)

$$\begin{array}{cc} \sum_{h=1}^{w}z_{gh}=1, & g\in R \end{array}$$ (14)

$$\begin{array}{cc} \left|\sum_{g=1}^{r}p_{g}z_{gh}-\sum_{g=1}^{r}p_{g}z_{gh^{\prime }}\right|\leq \lambda & h\neq h^{\prime },h,h^{\prime }\in W \end{array}$$ (15)

$$\begin{array}{cc} \left|\sum_{g=1}^{r}m_{gh}z_{gh}-\sum_{g=1}^{r}m_{gh}z_{gh^{\prime }}\right|\leq \tau & h\neq h^{\prime },h,h^{\prime }\in W \end{array}$$ (16)

$$\begin{array}{cc} \left|\sum_{g=1}^{r}S_{g}z_{gh}-\sum_{g=1}^{r}S_{g}z_{gh^{\prime }}\right|\leq 1 & h\neq h^{\prime },h,h^{\prime }\in W \end{array}$$ (17)

$$\begin{array}{cc} \left|\sum_{g=1}^{r}L_{g}z_{gh}-\sum_{g=1}^{r}L_{g}z_{gh^{\prime }}\right|\leq 1 & h\neq h^{\prime },h,h^{\prime }\in W \end{array}$$ (18)

Objective function (12) aims to maximise the ratio of service time to working time across all the mobile clinics which will result in the maximisation of workload balance. Constraint (13) ensures that each mobile clinic is assigned the same number of routes. Constraint (14) causes for each route to be assigned to a mobile, this will then be the same for every month to ensure continuity of care is maintained. Constraint (15) allows for even fairness in service times between all the mobiles, so that the difference between the total service times allocated to each mobile is below a certain constant, λ. Additionally, constraint (16) allows for even fairness in the total travel distance, so that the difference between the total travel distance allocated to each mobile is below a certain constant, *τ*. Constraint (17) result in the number of large farms assigned to each mobile to be evenly distributed, and constraint (18) does the same for the number of small farms. This means that between all the mobiles, the staff will have a very similar workload.

### 3.4 Stage 3

Stage 3 then looks at each individual mobile and creates a schedule with the routes that have been assigned to it in stage 2. A VRP was defined as it would ensure that the farms seen on consecutive days would be in a closer proximity, which would help the mobile staff keep track of their schedules, as well as allow for the mobiles to return to a farm the following day if necessary. A distance matrix is determined with the distances between routes to be the distances between the last farm of each route. Any consecutive large farms receive a penalty weight assigned to the corresponding entry in the distance matrix. A penalty of 100 is assigned to edges between large farms so as to prevent large farms from being seen on consecutive days. From this VRP, one route is designed that consists of all the routes assigned to the mobile in consideration. This route is then the scheduled for that mobile clinic, since it provides the order in which the routes must be serviced during any particular month.

#### 3.4.1 Stage 3 sets, parameters, variables and objective function

The model uses sets to define the structure of the problem, where

$$\begin{array}{ccc} C & \;\;\text{is}\;\text{the}\;\text{set}\;\text{of}\;\text{all}\;\text{routes,}\;\text{with}\;\text{a}\;\text{cardinality}\;\text{of}\;c, & \\ L & \;\;\text{is}\;\text{the}\;\text{set}\;\text{of}\;\text{depots,}\;\text{with}\;\text{a}\;\text{cardinality}\;\text{of}\;l, & \\ U & \;\;\text{is}\;\text{the}\;\text{set}\;\text{of}\;\text{all}\;\text{nodes,}\;\text{which}\;\text{includes}\;\text{all}\;\text{the}\;\text{routes}\;\text{as}\;\text{well}\;\text{as}\;\text{the}\;\text{depot,}\;\text{thus}\; & \\ & \;T=C\cup L,\text{which}\;\text{has}\;\text{a}\;\text{cardinality}\;\text{of}\;u=c+l,\;\text{and} & \\ G & \;\;\text{is}\;\text{the}\;\text{set}\;\text{of}\;\text{all}\;\text{edges}. \end{array}$$

The value for *c* is $\left\lceil \frac{r}{3}\right\rceil$, *l* is 1 and *u* is $\lceil \frac{r}{3}\rceil +1$. The model takes in several parameters as input which have been defined after running stage 2, where

$$\begin{array}{ccc} \alpha_{\mu \rho } & \;\;\text{is}\;\text{the}\;\text{travelling}\;\text{distance}\;\text{between}\;\text{routes}\;\mu \;\text{and}\;\rho ,\;\text{and} & \\ \beta_{\mu \rho } & \;\;\text{is}\;\text{the}\;\text{travelling}\;\text{time}\;\text{between}\;\text{route}\;\mu \;\text{and}\;\rho . \end{array}$$

The only variable defined for stage 3 is *w*_*μρ*_, which is used to indicate whether an edge (*μ*, *ρ*) is in the schedule. We can define

$$\begin{array}{c} w_{\mu \rho }=\{\begin{array}{cc} 1; & \text{if}\;\text{edge}\;\left(\mu ,\rho \right)\;\in G\;\text{is}\;\text{in}\;\text{the}\;\text{schedule}.\\ 0; & \text{otherwise}. \end{array} \end{array}$$

The objective function of stage 3 is to minimise the time that is spent travelling across all the routes created. This will ensure that the schedule created allows for no large farms to be seen on consecutive days and the consecutive routes will be in similar areas.

#### 3.4.2 Stage 3 formulation

With all the defined sets, variables, parameters, and the objective function, we can introduce the formulation, which is given by

$$\begin{array}{ccc} \text{Min}\;\sum_{\rho =1}^{u}\sum_{\mu =1}^{c} & \alpha_{\mu \rho }w_{\mu \rho }, & \end{array}$$ (19)

$$\begin{array}{cc} \text{subject}\;\text{to}:\;\sum_{\mu =1}^{u}w_{\mu \rho }=1, & \rho \in C \end{array}$$ (20)

$$\begin{array}{cc} \sum_{\rho =1}^{u}w_{\mu \rho }=1, & \mu \in C \end{array}$$ (21)

$$\begin{array}{cc} \sum_{\rho =1}^{c}w_{\mu \rho }\leq 1, & \mu \in L \end{array}$$ (22)

$$\begin{array}{cc} \sum_{\mu =1}^{c}w_{\mu \rho }\leq 1, & \rho \in L \end{array}$$ (23)

$$\begin{array}{cc} \sum_{\mu =1}^{u}w_{\mu \rho }=\sum_{\mu =1}^{u}w_{\rho \mu }, & \rho \in C \end{array}$$ (24)

$$\begin{array}{cc} w_{\mu \rho }=0, & \mu ,\rho \in L \end{array}$$ (25)

$$\begin{array}{cc} w_{\mu \rho }\in \{0,1\} & \mu ,\rho \in U \end{array}$$ (26)

Objective function (19) minimises the total routing cost, which is equivalent to minimising the travelling distance across the schedule. Constraints (20) and (21) ensure that each route is visited only once and then each mobile leaves the farm only once. Constraints (22) and (23) result in there being no route that does not originate from a depot. Constraint (24) ensures that there is route continuity, if a route is included at a point, it must leave that route before continuing. Constraint (25) results in there being no routes between the depots and constraint (26) ensures that *w*_*μρ*_ can only be 0 or 1.

### 3.5 Computer implementation

For the computer implementation of each of the above three stages, different software as well as variables, sets and parameters were used. Stage 1 is run using a VRPSolver framework [22]. VRPSolver is a Python package that is used to solve the VRP using a Branch-Cut-and-Price algorithm. Once stage 1 is complete, stage 2 is then run with these generated routes. Stage 2 is run in Lingo version 19.0. After stage 2 is completed, stage 3 uses the results from stage 2 to generate a schedule using VRPSolver in Python.

### 3.6 Phase 2 results

#### 3.6.1 Current routes

**Fig 3:**
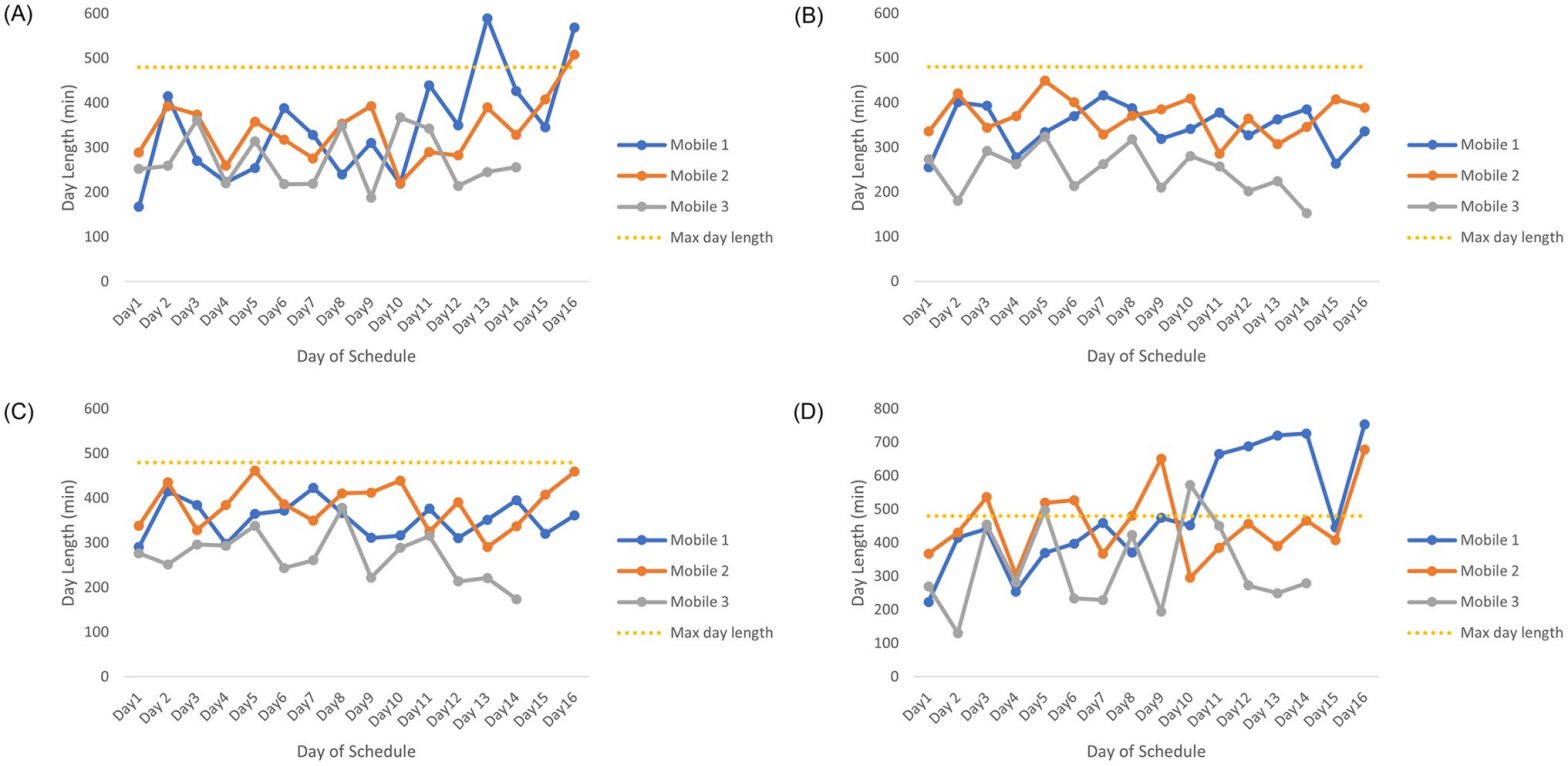
The day lengths for the current schedule with each set of service times. The lengths of the days for the current mobile schedule based on service times used in (A) E1, (B) E2, (C) E3 and (D) E4 and total travel time for the day, with the yellow dotted line indicating the maximum length of a working day.

Initially the current routes were investigated to compare with the new determined routes. Currently, mobile 1 and 2 have a 16-day schedule whereas mobile 3 has a 14-day schedule. The current schedules have the advantage that the routes scheduled within the same week are in a similar area. This has the benefit that if the mobile clinic needs to return to a farm, it is possible to do so after work has been completed the following day. The disadvantage to this is that for the routes to be in the same area, some days may be much shorter since there are no additional farms to see in this area. Contrary to this, there may be too many farms to see in a week, so the days are much longer. For the current routes, by using the four different determined service times, Fig 3 shows the expected lengths of the days for each of the mobile clinics for each day in the current monthly schedule, for each set of service times. All these service times produce large variances in the day lengths.

The total expected service time that is assigned to each mobile clinic for each set of service times is shown in Table 1. The total distance travelled by the mobile clinics over their 4-week schedule is shown in Table 1. Mobile 1 is assigned the largest service time across all sets of service times, for the current schedule. In the case of the E4 service times, mobile 1 is assigned almost double that of mobile 3. This highlights the uneven balance in the current schedule.

**Table 1:** Total service time (min) based on each of the four sets of service times, and total distance travelled for the full current schedules of the three mobile clinics.

Mobile name	Total service time (min)				Total distance travelled (km)
	E1	E2	E2	E4	
Mobile 1	4 844	4 855	4 964	7 163	695
Mobile 2	4 046	4 519	4 762	5 868	1350
Mobile 3	3 044	2 688	3 009	3 772	750

#### 3.6.2 Results from E1

**Fig 4:**
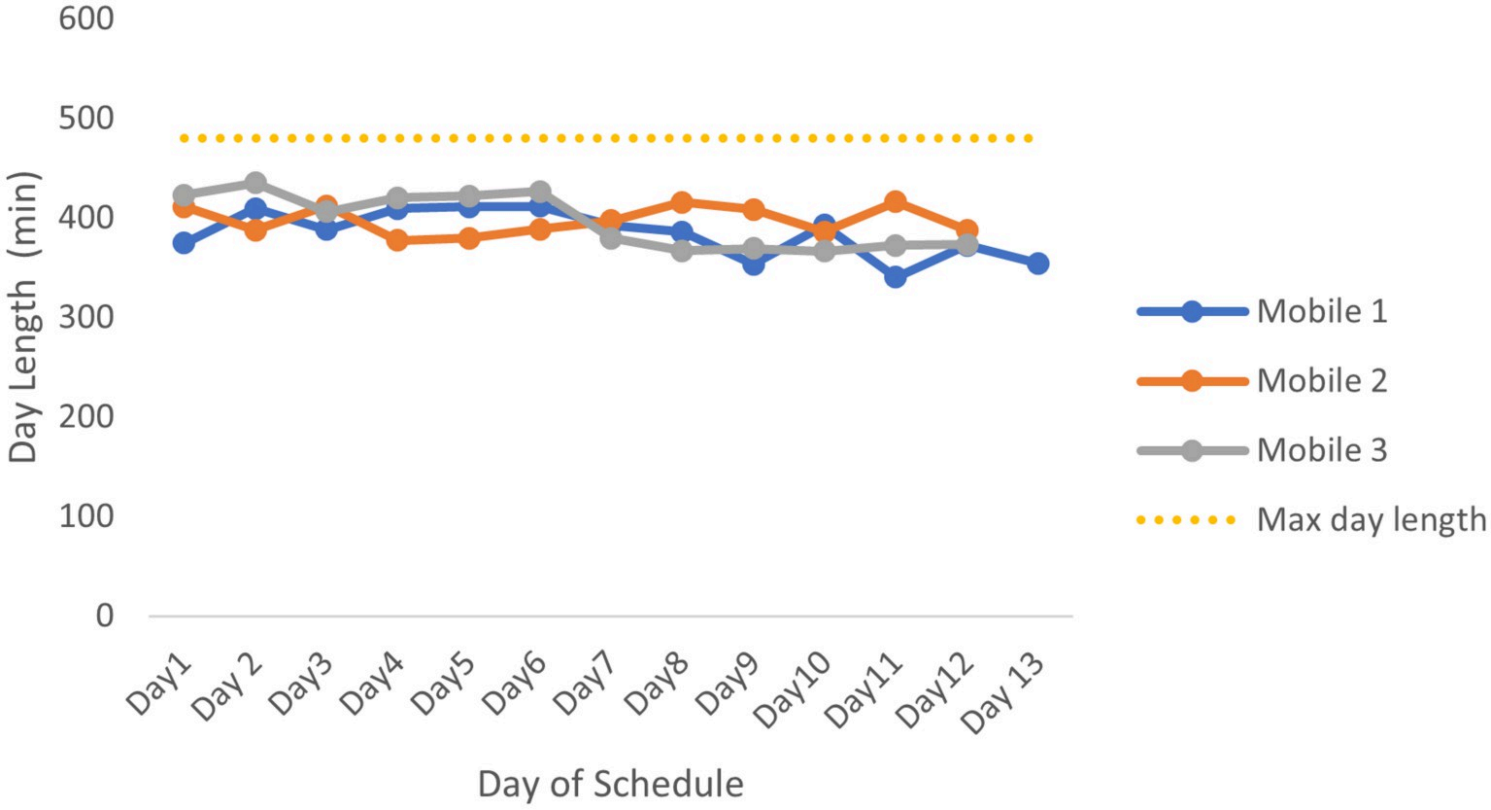
Day lengths for the E1 schedule for all 3 mobiles. The lengths of the days for the E1 mobile schedule consisting of E1 service times and total travel time for the day, with the yellow dotted line indicating the maximum length of a working day.

E1 was run using the average number of patients seen on a farm, multiplied by the average service time per patient as calculated using all farms and all mobile clinics over the 12 months of data received. An extra 10% of the calculated service times is added as a buffer. These times account for all factors, such as waiting on the farms and packing and unpacking of the mobile clinics. The expected lengths of the days of this schedule are shown in Fig 4. For this shorter schedule, the mobiles will leave the clinics at 08:00 and return between 13:45 and 15:15. Since admin takes approximately one hour per day, the staff will have enough time to complete their daily admin during this time. Fig 4 has minimal fluctuations, which is an indication that the workload between the mobiles and within the mobiles’ schedules is very similar.

Table 2 shows that the distribution of the service times and distances assigned to each mobile is very even. Each mobile sees more or less the same number of patients and travels the same distance throughout its whole schedule.

**Table 2:** Total service time (min) based on E1 service times, and total distance travelled for the full current and new schedule of the three mobile clinics.

Descriptors	Current schedule			E1 schedule		
	Mobile 1	Mobile 2	Mobile 3	Mobile 1	Mobile 2	Mobile 3
No. days on mobile	16	16	14	13	12	12
No. days in clinic	4	4	6	7	8	8
Total service time (min)	4 844	4 046	3 044	4 001	4 001	3 976
Total distance travelled (km)	695	1350	750	831	824	811

#### 3.6.3 Results from E2

E2 was obtained using the average length of time spent on a farm every month and adding extra buffer time of 10% of the calculated service times. These times account for all factors such as waiting on the farms and packing and unpacking of the mobile clinics.

**Fig 5:**
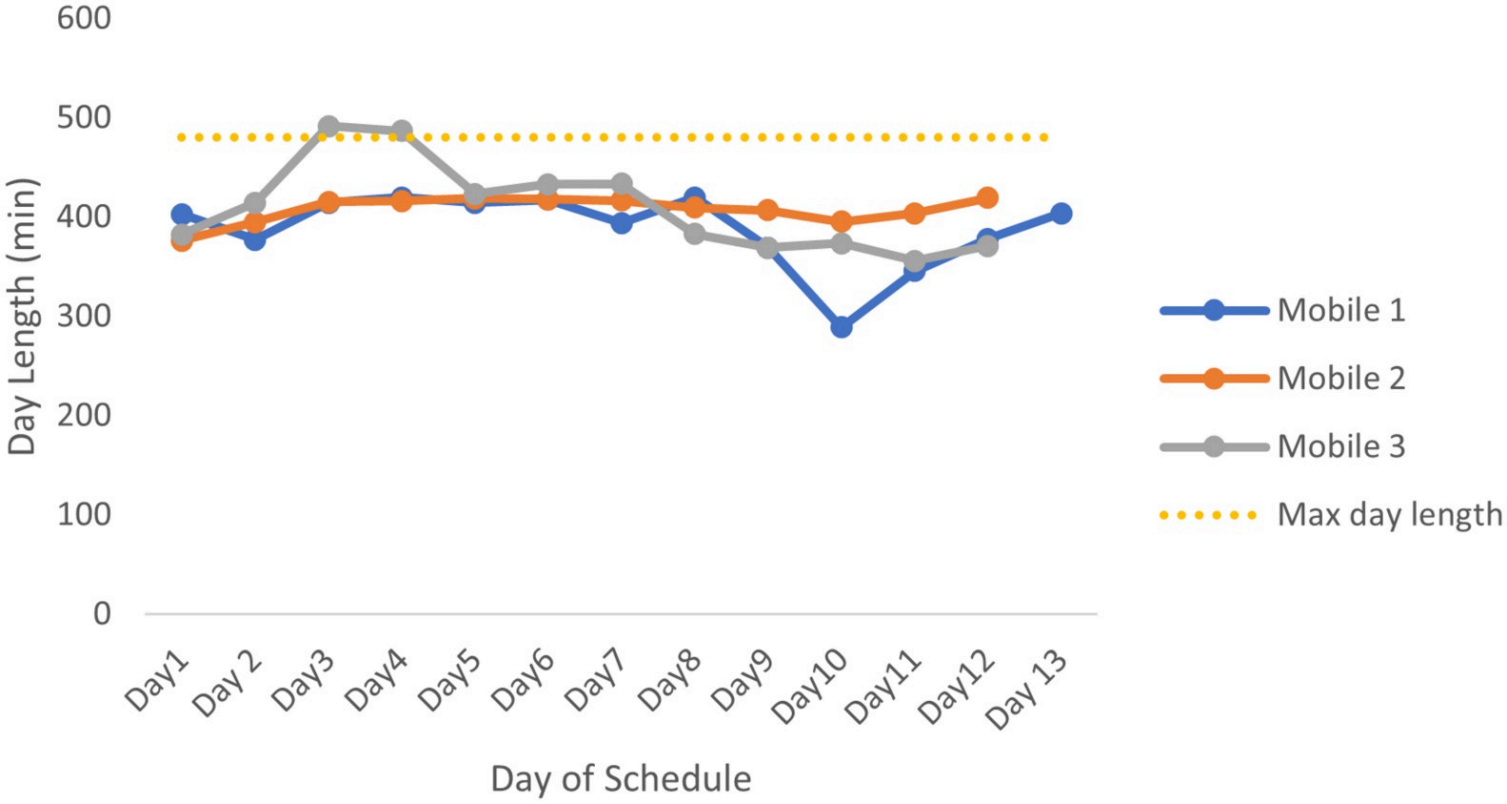
Day lengths for the E2 schedule for all 3 mobiles. The lengths of the days for the E1 mobile schedule consisting of E1 service times and total travel time for the day, with the yellow dotted line indicating the maximum length of a working day.

The expected day lengths for E2 are shown in Fig 5. Again there are minor fluctuations resulting in more balanced work days.

The distribution of the service time and distance travelled for each mobile in this schedule is even. Table 3 shows that each mobile clinic travels a similar total distance and sees a similar number of patients.

**Table 3:** Total service time (min) based on E2 service times, and total distance travelled for the full current and new schedule of the three mobile clinics.

Descriptors	Current schedule			E2 schedule		
	Mobile 1	Mobile 2	Mobile 3	Mobile 1	Mobile 2	Mobile 3
No. days on mobile	16	16	14	13	12	12
No. days in clinic	4	4	6	7	8	8
Total service time (min)	4 855	4 519	2 688	4 070	4 067	4 046
Total distance travelled (km)	695	1350	750	918	919	894

#### 3.6.4 Results from E3

The third schedule that was produced was run on service times that were calculated by taking the average length of time spent on a farm every month and adding a buffer time of the standard deviation of the time spent on each farm.

**Fig 6:**
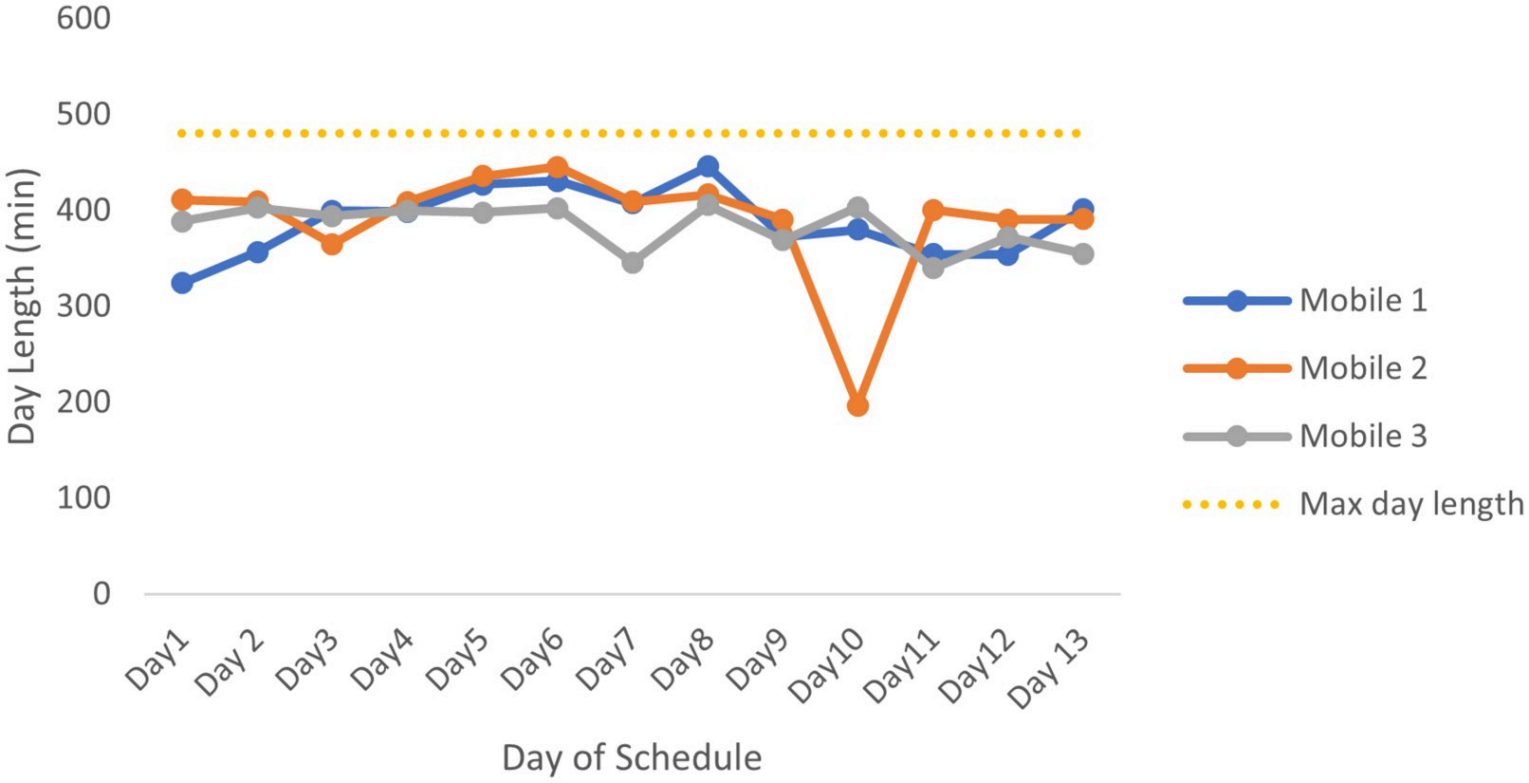
Day lengths for the E3 schedule for all 3 mobiles. The lengths of the days for the E3 mobile schedule consisting of E3 service times and total travel time for the day, with the yellow dotted line indicating the maximum length of a working day.

Fig 6 shows the lengths of the days of E3’s schedule. This graph has a similar structure to that of Fig 5, which should be expected since both schedules use similar service times. On mobile 2, the very short day was due to a day in which remaining farms had to be assigned to routes but there were not enough farms to fill the rest of the time. This shorter day can be used for additional admin, or support in the clinic. The rest of the graph shows minimal fluctuations, which is an indication that the workload is balanced between the mobiles and within the mobiles’ schedules. Table 4 shows the total service times and distance travelled for each mobile in this schedule, and for this schedule these are even.

**Table 4:** Total service time (min) based on E3 service times, and total distance travelled for the full current and new schedule of the three mobile clinics.

Descriptors	Current schedule			E3 schedule		
	Mobile 1	Mobile 2	Mobile 3	Mobile 1	Mobile 2	Mobile 3
No. days on mobile	16	16	14	13	13	13
No. days in clinic	4	4	6	7	7	7
Total service time (min)	4 964	4 762	3 009	4 263	4 264	4 242
Total distance travelled (km)	695	1350	750	714	730	715

#### 3.6.5 Results from E4

The fourth schedule produced was run on service times calculated as the maximum number of patients seen per month of the year and multiplied by the average service time per patient. This ensures that the patients should always get the time they require from the mobile clinics, even during months with seasonal workers.

**Fig 7:**
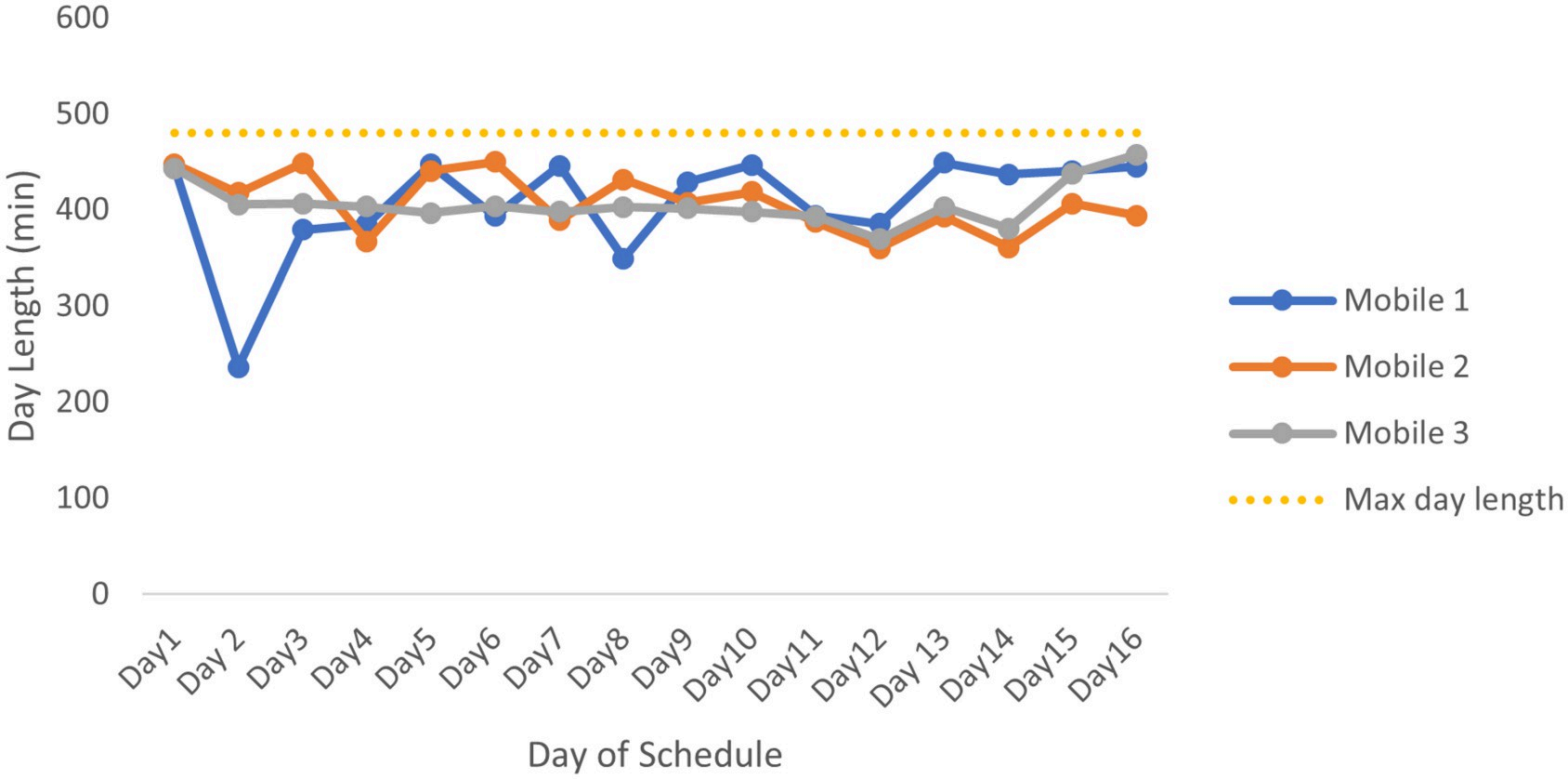
Day lengths for the E4 schedule for all 3 mobiles. The lengths of the days for the E4 mobile schedule consisting of E4 service times and total travel time for the day, with the yellow dotted line indicating the maximum length of a working day.

Fig 7 shows the lengths of the days of E4’s schedule. The day lengths are more or less the same with only one major fluctuation. Here we can see that on mobile 1 there is one very short day. The rest of the graph shows minimal fluctuations, which is an indication that the workload is balanced between the mobile clinics and within each mobile’s schedule.

Table 5 shows that the distribution of the service times and distances assigned to each mobile is very even. It can be seen that the service times for this schedule are much larger, which ensures that the patients can get the care required.

**Table 5:** Total service time (min) based on E3 service times, and total distance travelled for the full current and new schedule of the three mobile clinics.

Descriptors	Current schedule			E4 schedule		
	Mobile 1	Mobile 2	Mobile 3	Mobile 1	Mobile 2	Mobile 3
No. days on mobile	16	16	14	16	16	16
No. days in clinic	4	4	6	4	4	4
Total service time (min)	7 163	5 868	3 772	5 625	5 627	5 602
Total distance travelled (km)	695	1350	750	864	857	840

#### 3.6.6 Similarity in schedules

Since there will be a transition period, in which the staff move from the old schedule to the chosen new schedule, the staff may be interested in selecting a schedule that has the most farms assigned to the same mobiles as in the current schedule. This means that during the transition period the highest level of continuity of care should be maintained. Currently, mobile 1, mobile 2 and mobile 3 see 57, 52 and 39 farms respectively. Table 6 shows the percentage of similarity in the farms assigned to each mobile in the new schedule as compared to the current schedule. Table 6 also shows the number of farms for which their mobile assignment did not change from the current to the new schedule.

**Table 6:** The number of farms that have been assigned to the same mobile clinic as in the current schedules, as well as the total percentage similarity per schedule.

Schedule name	Number of farms in common			Total percentage similarity
	Mobile 1	Mobile 2	Mobile 3	
E1 Schedule	26	7	17	33.78%
E2 Schedule	17	7	6	20.27%
E3 Schedule	25	26	32	56.08%
E4 Schedule	25	29	32	58.11%

#### 3.6.7 Total distance travelled

The total distances travelled by each of the three mobile clinics across all their routes, was summed for the new schedules. These total distances are shown in Table 7.

**Table 7:** Total distance travelled for each schedule.

Schedule name	Total distance travelled (km)
Current schedule	2 750
E1 schedule	2 466
E2 schedule	2 731
E3 schedule	2 159
E4 schedule	2 561

## 4 Phase 3

In the third phase of the research, soft OR techniques were used with healthcare decision makers to analyse the results obtained from phase 2 and draw final conclusions. Final results and suggestions for improvement and implementation were then communicated with different stakeholders and healthcare professionals in the Cape Winelands district of the Department of Health, Western Cape, which includes the Witzenberg region, by means of an oral presentation, a written report, and two meetings held online and at the clinic.

### 4.1 Soft OR

Since four possible schedules were obtained in phase 2, each with its own advantages and disadvantages, the most suitable for the mobile clinics could not be determined without input from decision makers. The analytic hierarchy process (AHP) was used to determine which schedule should be chosen. Four different criteria were determined by investigating the advantages and disadvantages of each schedule, namely cost, robustness, extra time and continuity of care. A schedule that scores highly for the cost criterion, should have the lowest overall cost, thus the lowest total distance travelled by the mobile clinics. A schedule that has robustness is one that has space for the farm population numbers to grow in the future. Extra time ties into both having time for admin and working in the clinic. If the decision makers require more staff to work in the clinic, then a schedule with extra time will be preferred. The last criterion considered is continuity of care during the transition period. A schedule with a high similarity percentage as seen in Table 6, will be preferred.

#### 4.1.1 Weights of the alternatives

First the four schedules (alternatives) are compared for each of the four criteria, based on the quantitative results. While constructing all matrices, perfect consistency was maintained. Table 8 shows the pairwise comparisons for each alternative schedule based on cost. These pairwise comparison scores were determined based on how well the schedules performed in saving travelling cost, as obtained from the total distances in Table 7.

**Table 8:** The pairwise comparisons for each alternative based on cost.

	E1	E2	E3	E4
E1	1	$\frac{4}{3}$	$\frac{1}{6}$	$\frac{2}{3}$
E2	$\frac{3}{4}$	1	$\frac{1}{8}$	$\frac{1}{2}$
E3	6	8	1	4
E4	$\frac{3}{2}$	2	$\frac{1}{4}$	1

Each schedule was compared based on robustness by looking at the service times for the farms in each schedule. Since the E4 schedule’s service times were seen as an upper bound, this meant this schedule would perform the best in robustness. The other schedules had similar service times, and scaled accordingly. The robustness pairwise comparison values can be seen in Table 9.

**Table 9:** The pairwise comparisons for each alternative based on robustness.

	E1	E2	E3	E4
E1	1	$\frac{7}{8}$	$\frac{6}{8}$	$\frac{1}{8}$
E2	$\frac{8}{7}$	1	$\frac{6}{7}$	$\frac{1}{7}$
E3	$\frac{8}{6}$	$\frac{7}{6}$	1	$\frac{1}{6}$
E4	8	7	6	1

Extra time is seen as having extra days to complete admin and provide care in the clinic. To compare the schedules in terms of extra time, one can look at the length of each schedule. The E1 schedule is 37 days, over a 60-day period (4-weeks for three mobiles), this schedule allows for 23 days of extra time, thus it would score the highest in terms of extra time. However E4’s schedule is 48 days, allowing for the least amount of extra time. The extra time comparisons are given in Table 10.

**Table 10:** The pairwise comparisons for each alternative based on extra time.

	E1	E2	E3	E4
E1	1	1	3	8
E2	1	1	3	8
E3	$\frac{1}{3}$	$\frac{1}{3}$	1	$\frac{8}{3}$
E4	$\frac{1}{8}$	$\frac{1}{8}$	$\frac{3}{8}$	1

How well each of the schedules performs in terms of continuity of care can be seen in Table 6. Both the E3 and E4 schedule perform the best out of the schedules with over 50% similarity to the current schedule. However, E4 has a slightly higher percentage similarity compared to E3, thus its pairwise comparison value is slightly more than 1. The E2 schedule has the worst similarity percentage, resulting in it receiving the lowest comparison score when compared to all other schedules. Table 11 shows the resulting pairwise comparison matrix after these comparisons were performed.

**Table 11:** The pairwise comparisons for each alternative based on continuity of care.

	E1	E2	E3	E4
E1	1	3	$\frac{3}{5}$	$\frac{1}{2}$
E2	$\frac{1}{3}$	1	$\frac{1}{5}$	$\frac{1}{6}$
E3	$\frac{5}{3}$	5	1	$\frac{5}{6}$
E4	2	6	$\frac{6}{5}$	1

From the pairwise comparison matrices, the scores of each of the alternatives were determined. The *n*^th^ entry in this vector represents the score for the *n*^th^ alternative. For cost the score vector is $S_{Cost}=[\frac{4}{37},\;\frac{3}{37},\;\frac{24}{37},\;\frac{6}{37}]$. The scores for robustness are $S_{Robustness}=[\frac{21}{241},\;\frac{24}{241},\;\frac{28}{241},\;\frac{168}{241}]$. The scores for extra time are $S_{Extra\;time}=[\frac{24}{59},\;\frac{24}{59},\;\frac{8}{59},\;\frac{3}{59}]$. The continuity of care score vector is $S_{Cont.ofcare}=[\frac{1}{5},\;\frac{1}{15},\;\frac{1}{3},\;\frac{2}{5}]$.

#### 4.1.2 Weights of the criteria

A meeting with three healthcare decision makers from the Department of Health and Wellness, Cape Winelands District, were organised at the Department of Logistics, Stellenbosch University, to determine the importance of each main criterion. The data analysis, model development and results of the three-stage model, including the four different schedules, were presented with each of their advantages and disadvantages. From there the decision makers were allowed to investigate the routes and schedules and ask questions. Before AHP commenced, the three decision makers showed interest in the E3 schedule as it provided a 600km decrease in distance travelled by the mobile clinics and it allowed for extra time, enabling the mobile staff to work in the clinics on these days of the schedule.

It had not been made apparent until this discussion, that the clinics had just undergone major budget cuts from the Department of Health. This change in financial situation caused for the decision makers to approach this discussion with cost savings in mind, which was not expected. It was originally anticipated that a more robust schedule with higher continuity of care would be favoured, resulting in the E4 schedule to be most favourable.

In order to perform AHP, comparisons need to be determined for each decision maker for each of the criteria. From these discussions, it became clear quickly that the three decision makers had different priorities, related to the positions they hold within the Department of Health and Wellness.

Decision maker 1’s comparisons can be seen in Table 12. This decision maker is involved in top management for the entire district. Due to their position, cost and extra time in the local clinics were seen as much more important, with less regard for robustness and continuity of care of the mobile clinics. Decision maker 2 is directly involved as a healthcare professional with the management and service provided by the mobile clinics in the region. Their comparisons can be seen in Table 13. Due to their position, priorities lie with the optimal functioning of the mobile clinics. This resulted in more importance being placed on cost as well as the robustness of the schedules. Decision maker 3 is involved in the management of all the clinics in the region, this meant there was a focus on what would be beneficial for the clinics. The comparisons for this decision maker can be seen in Table 14. Due to the decrease in budget, this decision maker had a keen interest in having extra help in the clinics because there was a shortage of staff. Cost was also of high importance to this decision maker and they held little regard for continuity of care and robustness of the mobile clinics.

**Table 12:** The pairwise comparisons for the criteria of the schedules made by decision maker 1.

	C	R	E	CC
C	1	7	1	9
R	$\frac{1}{7}$	1	$\frac{1}{7}$	8
E	1	7	1	9
CE	$\frac{1}{9}$	$\frac{1}{8}$	$\frac{1}{9}$	1

Inconsistencies are highlighted in grey. C represents cost, R represents robustness, E represents extra time and CC represents continuity of care.

**Table 13:** The pairwise comparisons for the criteria of the schedules made by decision maker 2.

	C	R	E	CC
C	1	7	5	9
R	$\frac{1}{7}$	1	3	8
E	$\frac{1}{5}$	$\frac{1}{3}$	1	9
CC	$\frac{1}{9}$	$\frac{1}{8}$	$\frac{1}{9}$	1

Inconsistencies are highlighted in grey. C represents cost, R represents robustness, E represents extra time and CC represents continuity of care.

**Table 14:** The pairwise comparisons for the criteria of the schedules made by decision maker 3.

	C	R	E	CC
C	1	5	$\frac{1}{7}$	9
R	$\frac{1}{5}$	1	$\frac{1}{5}$	3
E	7	5	1	9
CC	$\frac{1}{9}$	$\frac{1}{3}$	$\frac{1}{9}$	1

Inconsistencies are highlighted in grey. C represents cost, R represents robustness, E represents extra time and CC represents continuity of care.

Each of the decision makers portrayed some level of inconsistency, which was identified by comparing the consistency index compared to the random index for decision maker’s pairwise comparison matrices. For acceptable inconsistencies, this value should be below 0.1, which was not true for any of the decision makers. The inconsistencies were identified and are highlighted in grey in their pairwise comparison matrices. These values were double checked with the decision makers, and altered accordingly to adjust for the inconsistencies and to allow for AHP to progress.

Thereafter, the weights for each of the criterion for each decision maker were determined. For decision maker 1, the weights are as follows *w* = [0.444, 0.060, 0.444, 0.053]. Multiplying these weights with the scores for each alternative provided in Section 4.1.1 resulted in the total score for each of the schedules for decision maker 1, they are as follows *s* = [0.244, 0.226, 0.373, 0.157]. E3 has the highest score at 0.373, therefore the E3 schedule should be selected for decision maker 1.

In the same manner, the weights for each criterion for decision maker 2 were determined as *w* = [0.428, 0.328, 0.208, 0.036]. Resulting in the total score for each of the schedules for decision maker 2 to be *s* = [0.167, 0.154, 0.356, 0.323]. For decision maker 2, E3 has the highest score of 0.356. Therefore, the E3 schedule should be selected for decision maker 2.

The weights for each criterion for decision maker 3 were determined as *w* = [0.429, 0.099, 0.429, 0.043]. The total score for each of the schedules for decision maker 3 are then *s* = [0.238, 0.222, 0.362, 0.178]. The highest score for decision maker 3 is for E3, which produces a score of 0.362. Therefore, the E3 schedule should be selected for decision maker 3.

All the decision makers had the highest score for E3, indicating that this schedule should be selected. This aligns with their initial interest in the E3 schedule. This also addresses the challenge to be able to create a solution that all stakeholders agree is optimal [2].

## 5 Discussion

Of the four schedules that were created, each had their own advantages and disadvantages. All four schedules were able to decrease the total distance travelled by the mobile clinics, with the E3 schedule reducing the distance travelled from 2 750km to 2 159km. Three of the four schedules allowed for extra days in the clinic, allowing extra help to be provided to the staff at the clinic. All the schedules had an even distribution of the service times and distance travelled by each of the mobile clinics, showing that a fair distribution of workload could be obtained between the mobiles.

All of the created schedules have an even distribution of the distance travelled and service times for each of the mobile clinics. Schedules with less days on the mobile allow for extra days of admin or clinical support in the base clinic. These extra days could also be used for future growth into new farms. However, if the service on the farms takes longer than anticipated, this could very easily result in the mobile working overtime. Schedules with more days on the mobile are more robust and allow for internal growth on the farms.

The current schedule has adjacent days with large routes, for instance on day 5 and day 6 of mobile 1’s schedule. This was amended in the new created schedules. The days that were identified to be large routes are spread evenly and not consecutively throughout the entire schedule, which allows for the staff to not become as fatigued. Each mobile in each schedule only has one large route difference between them, making this a fair distribution.

Using E3’s service times, which are slightly larger than both those of E1 and E2, the model was able to produce a schedule that has a lower total distance travelled. It decreased the total distance travelled by 26.5% compared to that of E2 and by 14.2% compared to E1. This schedule had a 22.6% reduction in travel distance as compared to the current schedule. In the E3 schedule, the total distance travelled across the three mobiles is the lowest of all the schedules, which can be seen from Table 7, thus this schedule is the most preferred from a cost savings perspective.

With E4’s large service times, that account for large numbers of patients seen on the farms, the model was still able to produce a 48-day schedule, showing that even in very busy months there is a way to see all the patients necessary without having to compromise on admin or having the need to return to farms to complete service, which is immediately an improvement from their current situation.

AHP was conducted with three key decision makers to determine which schedule would be most suitable based on their preferences. The results from AHP indicated that the E3 schedule should be chosen for each of the decision makers, based on their preferences of cost, robustness, extra time and continuity of care. Following after AHP, the staffs’ interests were brought into light. Due to the recent budget cuts that occurred in the clinic, the staff had a major focus on cost saving. This was highlighted in their preferences in AHP and for all three decision makers the E3 schedule was the most suited. Thus this schedule was recommended for implementation for the mobile clinics.

After performing AHP, slight alterations were made to the E3 schedule in order to create a schedule that satisfied all of the contextual constraints and priorities of the decision makers. This resulted in a schedule with a slightly less fair distribution, but still a substantial decrease in total distance travelled compared to the current schedule implemented. The importance of cultural insight and continuous communication with stakeholders were hence highlighted. Sometimes the optimal mathematical solution does not provide the best practical solution.

## 6 Conclusion

In this research project, the aim was two-fold: (1) to reduce implementation challenges by involving healthcare professionals in the different phases of the scientific process, and (2) to develop a model with which mobile clinics in the South African setting can be scheduled. The importance of having a champion on the inside, working on a critical issue, and gaining as much as possible contextual understanding and trust from healthcare professionals was demonstrated in the positive reception of results and suggestions for improvement. A mixed-methods approach in three phases of research not only facilitated better collaboration and communication between OR modellers and healthcare professionals, but also resulted in a solution well suited for the local context [23], and a willingness to implement. The routes and schedules obtained by the three-stage model provide an improvement in the fairness across the schedules while also decreasing the total distance travelled. The staff were able to choose a schedule that suited their needs and are currently in the process of implementing this schedule. Optimisation will improve efficiency of the system, and as a result either open additional slots to serve more farming communities, or extra time to help in the local clinics (i.e. improve access to healthcare in the rural communities) and contribute to increased job satisfaction by healthcare practitioners by having fairer and evenly spread schedules. Job satisfaction has been shown to result in better care provided to patients.

There are a few limitations that presented themselves throughout this project, most of which pertain to the data that was provided, highlighting the importance of data quality. The patient data that was provided was only about the number of patients that were seen on a day, irrespective of the fact that multiple farms could be visited in a day. These patients seen were not linked to a corresponding farm. Due to this data limitation, the average number of patients seen on a day was assumed to be the number of patients seen on a specific farm, and this was used in the model. However, it would be more appropriate to have information about the patients seen on each farm so that more accurate service times can be determined. Additionally, more farm specific data could result in specific healthcare interventions to be considered. For example, if there is a trend for a farm to have a higher proportion of patients seen (based on the total farm population) compared to other farms, it may be an indication that the population requires additional health intervention. In a similar light, another limitation was that the farms visited each day were not recorded, thus if the mobile clinics travelled to additional farms other than those indicated on the official schedule, there would be no record of the farms seen. The entire population in need of care on a large farm currently needs to be seen in one day. Splitting a large farm into 2 consecutive days might allow for less strain to be placed on the staff during these days and the patients can get the sufficient care required. Another idea would be to having two or more different schedules based on the season, which may allow for schedules that adapt to the change in populations. Currently, there is a preference for one schedule throughout the whole year since this ensures ease of use for the staff, which is why this was not addressed. Having a seasonal schedule would result in less need to work overtime during busy months and reduce the number of days that the staff return to the clinic early. It will ensure that patients always get the time they require from the mobile clinic, but also provide extra help in the local clinics during less busy months.

A final note on limitations: In a remote survey that was conducted, farm populations were only received for 39.86% of the farms. The staff should pursue receiving the rest of this data so that they can know the dominant age categories that make up each of the farms, which will aid them in preparing for the services required. In terms of modelling, service times could be calculated based on the structure of the population.
